# Fondaparinux versus Enoxaparina no Tratamento de Pacientes Obesos com Síndrome Coronariana Aguda

**DOI:** 10.36660/abc.20230793

**Published:** 2024-09-06

**Authors:** Beatriz Rocha Darzé, Carolina Costa da Silva Souza, Queila Borges de Oliveira, João Victor Santos Pereira Ramos, Mateus S. Viana, Eduardo Sahade Darzé, Luiz Eduardo Fonteles Ritt

**Affiliations:** 1 Escola Bahiana de Medicina e Saúde Pública Salvador BA Brasil Escola Bahiana de Medicina e Saúde Pública, Salvador, BA – Brasil; 2 Hospital Cárdio Pulmonar Instituto D'oxmlr de Pesquisa e Ensino Salvador BA Brasil Instituto D'or de Pesquisa e Ensino - Hospital Cárdio Pulmonar, Salvador, BA – Brasil

**Keywords:** Anticoagulantes, Enoxaparina, Fondaparinux, Obesidade, Síndrome Coronariana Aguda

## Abstract

**Fundamento:**

O fondaparinux é um anticoagulante eficaz e seguro usado no tratamento de síndromes coronarianas agudas (SCAs). No entanto, devido à baixa representatividade de indivíduos obesos em ensaios clínicos, os efeitos de se aplicar os resultados desse medicamento nesta população continuam incertos.

**Objetivos:**

Comparar o fondaparinux à enoxaparina no tratamento de obesos com SCA.

**Métodos:**

Este é um estudo do tipo coorte retrospectivo, incluindo indivíduos obesos (IMC ≥ 30 Kg/m^2^) internados com Infarto do Miocárdio sem Elevação do Segmento ST (IAMSSST) ou Angina Instável (AI) e tratados com fondaparinux ou enoxaparina entre 2010 e 2020. Os grupos que receberam fondaparinux e enoxaparina foram comparados quanto suas características clínicas e laboratoriais usando o teste do qui-quadrado e o teste de Mann-Whitney, conforme apropriado. A incidência dos desfechos primários (morte, reinfarto, acidente vascular cerebral, sangramento maior) foi comparada entre os grupos. Um p<0,05 foi considerado estatisticamente significativo em todas as análises.

**Resultados:**

Um total de 367 pacientes obesos com IAMSSST ou AI foi incluído, dos quais 258 usaram fondaparinux e 109 usaram enoxaparina. A idade média foi 64 ± 12 anos, 52,9% eram do sexo masculino. A prevalência e diabetes, hipertensão, dislipidemia, doença arterial coronariana prévia, acidente vascular cerebral prévio, e implementação de estratégia invasiva foi similar entre os grupos. A incidência do desfecho primário foi 4,7% no grupo fondaparinux e 5,5% no grupo enoxaparina (p = 0,729). Não houve diferença entre os grupos quando os componentes do desfecho primário foram analisados separadamente.

**Conclusão:**

Em uma amostra de pacientes obesos com IAMSSST ou AI, não houve diferença na ocorrência do desfecho composto (morte, acidente vascular cerebral, reinfarto, sangramento maior) entre os pacientes que utilizaram fondaparinux ou enoxaparina.

**Figure:**
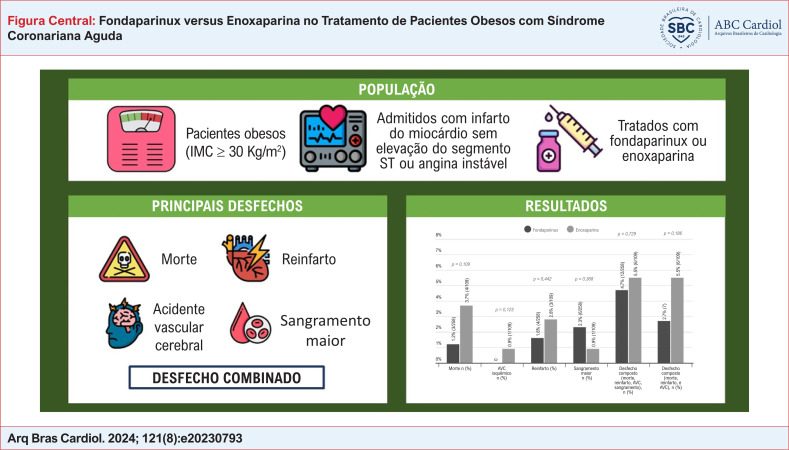


## Introdução

As síndromes coronarianas agudas (SCA) são a principal causa de morte no Brasil e no mundo.^1^ A taxa de sobrevida após um evento coronariano agudo aumentou substancialmente nas últimas décadas, devido aos avanços nas terapias medicamentosas e de reperfusão.^2,3^ Contudo, esses pacientes ainda apresentam risco de desenvolverem desfechos adversos como morte, reinfarto, acidente vascular cerebral e sangramento.^4^

A terapia anticoagulante é uma das etapas mais importantes no tratamento inicial das SCA. A enoxaparina é tradicionalmente administrada com titulação da dose baseada no peso, enquanto o fondaparinux tem dose fixa.^5^ Os estudos OASIS-5 e OASIS-6 compararam esses medicamentos em pacientes com SCA e demonstraram que o fondaparinux está associado a um melhor desfecho, principalmente pela redução de sangramentos.^6,7^ Contudo, a farmacocinética e a farmacodinâmica dos medicamentos podem se diferir entre pacientes obesos e não obesos,^8^ e o fato de populações obesas serem historicamente pouco representadas em ensaios clínicos leva a incertezas quanto à aplicação desses resultados a pacientes com um IMC ≥ 30 kg/m^2^.

Dada a elevada prevalência da obesidade na população global e sua associação com um risco aumentado de Doença Arterial Coronariana (DAC), estabelecer um regime de tratamento antitrombótico apropriado para esses pacientes é de extrema importância. Assim, o objetivo deste estudo foi comparar o fondaparinux e a enoxaparina no tratamento das SCA em uma população de pacientes obesos.

## Métodos

### Delineamento do estudo

Este foi um estudo retrospectivo de pacientes hospitalizados com SCA em um hospital cardíaco em Salvador, Bahia, Brasil.

### População do estudo, critérios de inclusão e de exclusão

Foram incluídos pacientes com um IMC ≥ 30 Kg/m^2^, com idade superior a 18 anos, internados no hospital com um diagnóstico de Angina Instável (AI) ou Infarto do Miocárdio Sem Elevação do Segmento ST (IAMSSST), e tratados com fondaparinux ou enoxaparina entre 2010 e 2020. Foram excluídos pacientes transferidos para ou de outro hospital com Infarto do Miocárdio (IM) tipo II, ou quando SCA foram excluídas.

De acordo com o protocolo institucional, a enoxaparina na dose de 1 mg por quilo de peso corporal a cada 12 horas era o antitrombótico de escolha até 2012. Entre 2012 e 2019, fondaparinux na dose de 2,5 mg por dia era indicada. Após 2018, devido aos baixos estoques de fondaparinux, a enoxaparina voltou a ser utilizada rotineiramente.

Pacientes com disfunção renal (*clearance de creatinina*, ClCr < 30mL/min/1,73 m^2^) receberam uma dose reduzida de enoxaparina de 1 mg/Kg/dia. Pacientes com peso acima de 100 Kg receberam a dose máxima de enoxaparina de 100mg, 12 em 12 horas; a dose de fondaparinux continuou 2,5 mg/dia.

### Procedimentos do estudo

Dados clínicos e demográficos, exames complementares, e desfechos primários de todos os pacientes com SCA foram coletados continuamente e prospectivamente coletados como parte de um programa de assistência de qualidade na SCA na instituição durante sua internação. Esses dados foram acessados retrospectivamente para o estudo.

### Considerações éticas

O protocolo foi aprovado pelo Comitê de Ética Professor Celso Figueirôa do Hospital Santa Izabel (Santa Casa de Misericórdia da Bahia) sob o número 3.725.420. Uma vez que este foi um estudo retrospectivo baseado em dados coletados de prontuários médicos e relatórios de controle de qualidade, solicitou-se isenção do termo de consentimento, o que foi aceito pelo comitê de ética.

### Desfechos clínicos

O desfecho primário consistiu em mortalidade por todas as causas, reinfarto (com base na 4ª definição universal de IM),^9^ acidente vascular cerebral e sangramento maior durante a internação. Acidente vascular cerebral foi definido como presença de um novo déficit neurológico focal, de origem vascular, com sinais ou sintomas com duração de mais de 24 horas. Sangramento importante baseou-se nos seguintes critérios: Hb/Ht ≤ 2g/dL, transfusão de sangue, ou instabilidade hemodinâmica. As definições foram consistentes com as utilizadas no estudo OASIS-5.^6^

### Análise estatística

Os dados foram processados usando o *Statistical Package for the Social Sciences* (SPSS 25.0). As variáveis categóricas foram descritas como porcentagens ou proporções e comparadas usando o teste do qui-quadrado, enquanto as variáveis contínuas, por apresentarem distribuição normal, foram expressas como média e Desvio Padrão (DP) e comparadas usando o teste t de *Student* não pareado. A normalidade dos dados foi verificada pelo teste de Kolmogorov-Smirnov e análise visual por histograma. O nível de significância adotado foi de 5%.

A análise de sensibilidade foi realizada com os pacientes que se submeteram à angiografia coronária. Nós consideramos esse subgrupo de pacientes para a análise de sensibilidade, devido ao risco de sangramento nessa população.

## Resultados

**Figura 1:**
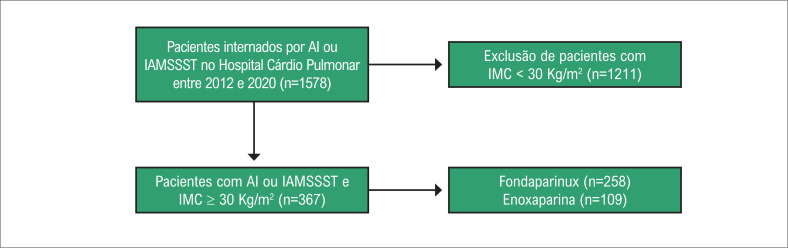
Fluxograma da seleção dos pacientes; IMC: índice de massa corporal; IAMSSST: infarto agudo do miocárdio sem elevação do segmento ST; AI: angina instável.

Entre 1578 pacientes internados por SCA, 367 pacientes obesos com IAMSSST ou AI foram incluídos, dos quais 259 usaram fondaparinux e 109 usaram enoxaparina (Figura 1).

Na população geral, a idade média foi 64 ± 12, 52,9% eram homens. Não foram observadas diferenças significativas entre os grupos que receberam fondaparinux e enoxaparina em termos de características clínicas, como mostrado na Tabela 1. Somente a fração de ejeção foi mais alta no grupo enoxaparina (63 ± 11%) em comparação ao grupo fondaparinux (60 ± 13%; p < 0,05), mas ambos estavam dentro da faixa de normalidade. O IMC foi similar entre os grupos (33,7 ± 4,0 Kg/m^2^ no fondaparinux versus 33,6 ± 3,5 Kg/m^2^ no enoxaparina). Estratégia invasiva foi realizada em 275 pacientes (74.9%), sem diferença entre os grupos fondaparinux (74,0%) e enoxaparina (77,1%) (p = 0,54).

**Tabela 1:** Características gerais da população do estudo

	Total (n = 367)	Fondaparinux (n = 258)	Enoxaparin (n = 109)	Valor de p
Idade (±SD)	64,5 (±12,4)	63,7 (±12,1)	66,3 (±13,0)	0,08
Tipo de SCA				
- Angina instável; n (%)	214 (58,3%)	155 (60,1%)	59 (54,1%)	0,29
- IAMSSST, n (%)	153 (41,7%)	103 (39,9%)	50 (45,9%)	0,29
Sexo masculino, n (%)	194 (52,9%)	135 (52,3%)	59 (54,1%)	0,75
Diabetes Mellitus n (%)	162 (44,1%)	115 (44,6%)	47 (43,1%)	0,80
Hipertensão, n (%)	312 (85,0%)	220 (85,3%)	92 (84,4%)	0,83
Dislipidemia, n (%)	238 (64,9%)	164 (63,6%)	74 (67,9%)	0,43
Tabagismo, n (%)	27 (7,4%)	18 (7,0%)	9 (8,3%)	0,67
ICC prévia, n (%)	16 (4,4%)	8 (3,1%)	8 (7,3%)	0,07
DAP prévia, n (%)	5 (1,4%)	2 (0,8%)	3 (2,8%)	0,14
DAC prévia, n (%)	144 (39,2%)	95 (36,8%)	49 (45,0%)	0,15
ICP prévia, n (%)	60 (16,3%)	38 (14,8%)	22 (20,2%)	0,16
CABG prévio, n (%)	69 (18,8%)	45 (17,5%)	24 (22%)	0,32
AVC prévio n (%)	14 (3,8%)	10 (3,9%)	4 (3,7%)	0,93
Killip 1 n (%)	345 (83,9%)	232 (82,6%)	113 (86,9%)	0,26
Medicamentos				
- Aspirina, n (%)	349 (95,1%)	248 (96,1%)	101 (92,7%)	0,16
- Inibidores de receptor P2Y12, n (%)	340 (92,6%)	243 (94,2%)	97 (89,0%)	0,08
- Abciximabn (%)	5 (1,4%)	4 (1,6%)	1 (0,9%)	0,63
- Tirofibana n (%)	1 (0,3%)	0 (0,0%)	1 (0,9%)	0,12
- NACO n (%)	11 (3,0%)	5 (1,9%)	6 (5,5%)	0,07
- Varfarina n (%)	7 (1,9%)	3 (1,2%)	4 (3,7%)	0,11
Estratégia invasiva, n (%)	275 (74,9%)	191 (74,0%)	84 (77,1%)	0,54
ICP n (%)	92 (25,9%)	66 (26,4%)	26 (24,5%)	0,66
Acesso radial, n (%)	220 (59,9%)	151 (58,5%)	69 (63,3%)	0,39
PAS média (±DP) mmHg	145,6 (±25,4)	146,9 (±25,8)	142,6 (±24,3)	0,14
FC média (±SD) bpm	74,3 (±16,4)	73,5 (±15,4)	76,0 (±18,5)	0,19
Creatinina média (±SD) mg/dL	1,0 (±0,34)	0,99 (±0,32)	1,03 (±0,39)	0,23
Clearance de creatinina (±SD) mL/min/1,73m²	98,3 (±29,2)	99,8 (±28,3)	94,7 (±31,2)	0,12
Fração de Ejeção Média (±DP) %	0,62 (±0,11)	0,63 (±0,11)	0,60 (±0,13)	0,02
Hb média (±DP) mg/dL	13,6 (±1,5)	13,6 (±1,5)	13,5 (±1,6)	0,65
Ht médio (±DP) %	41,0 (±4,4)	41,1 (±4,3)	40,8 (±4,6)	0,49
IMC médio (±DP) Kg/m^2^	33.7 (±3,8)	33.7 (±4,0)	33.6 (±3,5)	0,81
Tempo médio de internação (±DP) (dias)	6.1 (±6,5)	5,8 (6,4)	6,8 (6,7)	0,18

SCA: síndrome coronariana aguda; IAMSSST: infarto do miocárdio sem elevação do segmento ST; ICC: insuficiência cardíaca congestiva; DAP: doença arterial periférica; DAC: doença arterial coronariana; AVC: acidente vascular cerebral; NACO: novos anticoagulantes orais; PAS: pressão arterial sistólica; FC: frequência cardíaca; DP: desvio padrão; Hb: hemoglobina. Ht: hematócrito; IMC: índice de massa corporal; ICP: intervenção coronária percutânea; CABG: bypass da artéria coronária.

**Figura 2:**
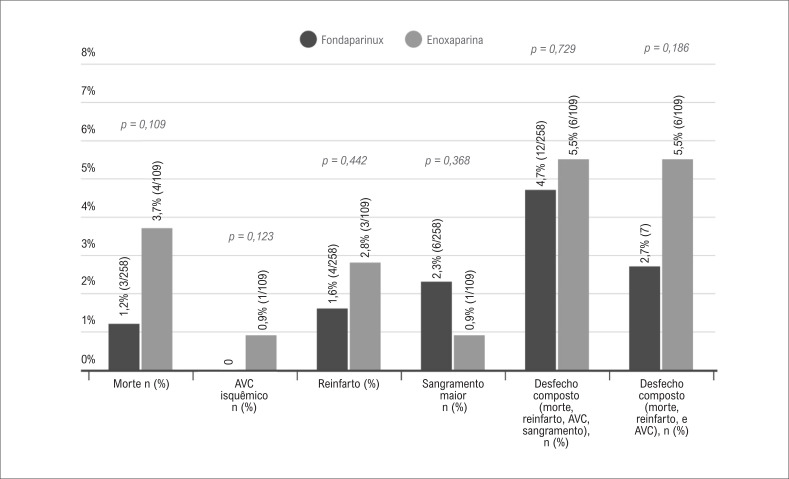
Incidência de desfechos primários na população do estudo; AVC: acidente vascular cerebral.

A incidência do desfecho primário (morte, reinfarto, acidente vascular cerebral ou sangramento maior) foi similar entre os grupos: 4,7% no grupo fondaparinux, e 5,5% no grupo enoxaparina (p=0,729) (Figura 2). Ainda, não houve diferença significativa entre os grupos quanto aos componentes do desfecho secundário, tanto quando analisados juntos como quando analisados individualmente.

**Figura 3:**
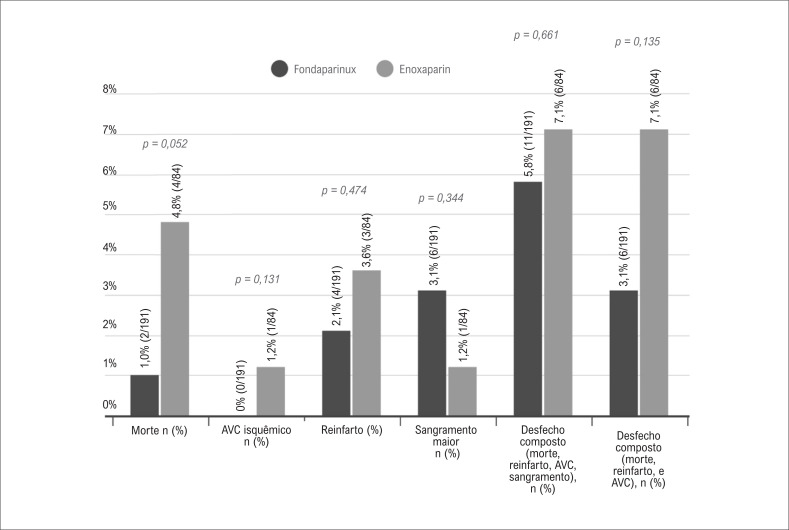
Incidência de desfechos primários em pacientes que se submeteram à angiografia coronária; AVC: acidente vascular cerebral.

A análise de sensibilidade está apresentada na Figura 3. A incidência de desfecho primário composto permaneceu similar entre os grupos quando analisados somente aqueles pacientes que se submeteram à estratégia invasiva: 5,8% no grupo fondaparinux e 7,1% no grupo enoxaparina (p = 0,661). Também não se observou diferença significativa entre os grupos quanto ao desfecho composto por morte, reinfarto ou acidente vascular cerebral (p = 0,135), ou quando os desfechos foram analisados individualmente (Figura 3).

## Discussão

Com base em um estudo retrospectivo do tipo coorte de pacientes admitidos por AI ou IAMSSST, nosso estudo comparou fondaparinux com enoxaparina no tratamento de pacientes obesos. Não foi observada diferença na incidência de morte, reinfarto, acidente vascular cerebral ou sangramento maior entre os dois subgrupos. Levantou-se a hipótese de que o fondaparinux seja tão eficaz e seguro como a enoxaparina, mesmo em pacientes obesos, sem a necessidade de ajuste da dose.

O estudo OASIS-5^6^ avaliou uma amostra de 20 078 pacientes com IAMSSST aleatoriamente separados para receberem fondaparinux ou enoxaparina, e identificou equivalência entre as estratégias no desfecho primário (morte ou infarto do miocárdio ou isquemia refratária em nove dias). Identificou-se uma redução na ocorrência de sangramento maior nos pacientes utilizando fondaparinux (2,2% versus 4,1%, RR 0,52; p < 0,001), resultando em uma redução na mortalidade de 30 dias a favor do fondaparinux. No entanto, o estudo não avaliou especificamente pacientes com IMC ≥ 30 Kg/m^2^, levantando a questão de se a dose fixa de 2,5mg de fondaparinux teria um desempenho similar em pacientes nessa faixa de IMC.

Soeiro et al.^10^ conduziram um estudo coorte multicêntrico retrospectivo incluindo 2282 pacientes e compararam fondaparinux versus enoxaparina com base em um registro brasileiro. Similar aos estudos OASIS, o fondaparinux mostrou superioridade à enoxaparina para a população brasileira, com redução significativa na ocorrência de sangramentos e eventos combinados (choque cardiogênico, IM, morte, acidente vascular cerebral isquêmico, e sangramento maior). Não houve diferença no desfecho primário de mortalidade por todas as causas entre os dois grupos.

Em nossa análise, não houve diferença significativa na incidência de sangramentos entre pacientes obesos tratados com fondaparinux e enoxaparina. Diferentemente do que foi encontrado em estudos anteriores, observou-se uma tendência a uma taxa mais alta de sangramentos no grupo fondaparinux (2,3% vs. 0,9%). O fato de que essa diferença não tenha sido estatisticamente significativa (p=0,368) pode estar relacionado ao poder reduzido de se analisar esse desfecho isoladamente. Em nosso conhecimento, não há nenhum outro estudo na literatura avaliando a segurança e a eficácia do fondaparinux em pacientes obesos com SCA sem elevação do segmento ST. Ainda no contexto da SCA, Spinler et al.^11^ conduziram uma análise de subgrupos (obesos vs. não obesos) baseada nos dados combinados dos ensaios clínicos ESSENCE e TIMI-11B. Esses estudos randomizados compararam enoxaparina e Heparina Não Fracionada (HNF) para o tratamento de SCA. O desfecho primário usado na análise combinada foi o desfecho composto por morte, IM, e revascularização urgente, e a superioridade da enoxaparina sobre a HNF foi observada tanto em obesos como em não obesos. Embora este estudo tenha diferentes intervenções e comparações, o resultado favorável na população obesa corrobora nosso presente estudo, sugerindo que não há necessidade de ajuste à terapia nos pacientes obesos.

Davidson et al.,^12^ com base nos dados do ensaio MATISSE, compararam o uso do Fondaparinux ao tratamento clássico com heparinas (enoxaparina ou HNF) em pacientes obesos e não obesos com tromboembolismo venoso (trombose venosa profunda), e demonstraram que, independentemente do IMC, não foram observadas diferenças na taxa de sangramentos entre os grupos que receberam fondaparinux e heparinas. Embora este estudo tenha sido conduzido em uma população diferente (tromboembolismo venoso), esses achados corroboram nossos resultados, sugerindo que o IMC ≥ 30 Kg/m^2^ tenha pouco ou nenhum impacto sobre a segurança e a eficácia da terapia anticoagulante. Assim, nossos dados dão força ao uso do fondaparinux em sua dose habitual para SCA (2,5mg) em pacientes com IMC ≥ 30 Kg/m^2^.

Este estudo é pioneiro em comparar desfechos clínicos relacionados ao uso do fondaparinux ou da enoxaparina em pacientes obesos com SCA. No entanto, algumas limitações metodológicas devem ser destacadas, principalmente a natureza observacional e retrospectiva do estudo, embora as características clínicas das populações tenham sido similares. Análise com escores de propensão poderiam ser usados em estudos como o nosso; contudo, como as características basais estavam bem equilibrados entre os grupos, escolhemos não a realizar. Além disso, uma vez que a população era composta de pacientes obesos, o tamanho de nossa amostra era pequeno, o que pode haver mascarado diferenças eventuais entre os grupos. Além disso, no desfecho clínico, pudemos avaliar somente morte por todas as causas, e seria interessante separá-la de morte cardiovascular.

Ainda, acreditamos que nosso estudo contribui de maneira significativa na geração de hipóteses, e estudos randomizados nesta população são necessários para confirmar nossos achados. Seriam importantes estudos com uma coorte de pacientes com SCA sem elevação de ST utilizando fondaparinux, que incluam a comparação da eficácia e da segurança do medicamento em obesos e não obesos.

## Conclusões

Em uma amostra de pacientes com IMC ≥ 30 Kg/m^2^ e SCA sem elevação do segmento ST, o tratamento antitrombótico com fondaparinux ou enoxaparina não se associou com diferenças na ocorrência de eventos cardiovasculares maiores ou sangramento maior durante o período de internação.

## References

[1] World Health Organization (2020). The Top 10 Causes of Death, 2000-2019 [Internet].

[2] Heidenreich PA, McClellan M (2001). Trends in Treatment and Outcomes for Acute Myocardial Infarction: 1975-1995. Am J Med.

[3] Laforgia PL, Auguadro C, Bronzato S, Durante A (2022). The Reduction of Mortality in Acute Myocardial Infarction: From Bed Rest to Future Directions. Int J Prev Med.

[4] Brieger D, Fox KA, Fitzgerald G, Eagle KA, Budaj A, Avezum A (2009). Predicting Freedom from Clinical Events in non-ST-elevation Acute Coronary Syndromes: The Global Registry of Acute Coronary Events. Heart.

[5] Byrne RA, Rossello X, Coughlan JJ, Barbato E, Berry C, Chieffo A (2023). 2023 ESC Guidelines for the Management of Acute Coronary Syndromes. Eur Heart J.

[6] Yusuf S, Mehta SR, Chrolavicius S, Afzal R, Pogue J, Granger CB (2006). Comparison of Fondaparinux and Enoxaparin in Acute Coronary Syndromes. N Engl J Med.

[7] Yusuf S, Mehta SR, Chrolavicius S, Afzal R, Pogue J, Granger CB (2006). Effects of Fondaparinux on Mortality and Reinfarction in Patients with Acute ST-segment Elevation Myocardial Infarction: The OASIS-6 Randomized Trial. JAMA.

[8] Badimon L, Vera RH, Padró T, Vilahur G (2013). Antithrombotic Therapy in Obesity. Thromb Haemost.

[9] Thygesen K, Alpert JS, Jaffe AS, Chaitman BR, Bax JJ, Morrow DA (2019). Fourth Universal Definition of Myocardial Infarction (2018). Eur Heart J.

[10] Soeiro AM, Silva PG, Roque EA, Bossa AS, César MC, Simões SA (2016). Fondaparinux versus Enoxaparin - Which is the Best Anticoagulant for Acute Coronary Syndrome? - Brazilian Registry Data. Arq Bras Cardiol.

[11] Spinler SA, Inverso SM, Cohen M, Goodman SG, Stringer KA, Antman EM (2003). Safety and Efficacy of Unfractionated Heparin versus Enoxaparin in Patients who are Obese and Patients with Severe Renal Impairment: Analysis from the ESSENCE and TIMI 11B Studies. Am Heart J.

[12] Davidson BL, Büller HR, Decousus H, Gallus A, Gent M, Piovella F (2007). Effect of Obesity on Outcomes after Fondaparinux, Enoxaparin, or Heparin Treatment for Acute Venous Thromboembolism in the Matisse trials. J Thromb Haemost.

