# Supplementary material for: Fondaparinux versus Enoxaparina no Tratamento de Pacientes Obesos com Síndrome Coronariana Aguda
Source: Arq Bras Cardiol. 2024 Sep 6;121(8):e20230793. [Article in Portuguese] doi: 10.36660/abc.20230793 (PMC11495804; doi:10.36660/abc.20230793)
Supplement: Translation

# Translation: Fondaparinux versus Enoxaparin in the Treatment of Obese Patients with Acute Coronary Syndrome

**DOI:** 10.36660/abc.20230793i

**Authors:** Beatriz Rocha Darzé, Carolina Costa da Silva Souza, Queila Borges de Oliveira, João Victor Santos Pereira Ramos, Mateus S. Viana, Eduardo Sahade Darzé, Luiz Eduardo Fonteles Ritt

**Affiliations:** 1 Salvador BA Brazil Escola Bahiana de Medicina e Saúde Pública, Salvador, BA – Brazil; 2 Salvador BA Brazil Instituto D'or de Pesquisa e Ensino - Hospital Cárdio Pulmonar, Salvador, BA – Brazil

**Keywords:** Anticoagulants, Enoxaparin, Fondaparinux, Obesity, Acute Coronary Syndrome

## Abstract

**Background:**

Fondaparinux is an effective and safe anticoagulant in the treatment of acute coronary syndromes (ACS). However, due to the low representation of obese individuals in clinical trials, the effects of applying the results of this drug to this population remain uncertain.

**Objectives:**

To compare Fondaparinux to Enoxaparin in the treatment of obese patients with ACS.

**Methods:**

This is a retrospective cohort study, including obese individuals (BMI ≥ 30 Kg/m^2^) admitted with non-ST-segment elevation myocardial infarction (NSTEMI) or unstable angina (UA) and treated with Fondaparinux or Enoxaparin between 2010 and 2020. The Fondaparinux and Enoxaparin groups were compared for their clinical and laboratory characteristics using chi-square and Mann-Whitney tests, as appropriate. The incidence of primary outcomes (death, reinfarction, stroke, major bleeding) was compared between groups. P-value < 0.05 was considered significant for all analyses.

**Results:**

A total of 367 obese patients with NSTEMI or UA were included, of whom 258 used Fondaparinux and 109 used Enoxaparin. Mean age was 64 ± 12 years, and 52.9% were male. The prevalence of diabetes, hypertension, dyslipidemia, prior coronary artery disease, prior stroke, and implementation of invasive strategy was similar between groups. The incidence of the primary outcome was 4.7% in the Fondaparinux group and 5.5% in the Enoxaparin group (p = 0.729). There was no difference between groups when analyzing the components of the primary outcome separately.

**Conclusion:**

In a sample of obese patients with NSTEMI or UA, there was no difference in the occurrence of the composite outcome (death, stroke, reinfarction, major bleeding) between patients who used Fondaparinux or Enoxaparin.

## Introduction

Acute coronary syndromes (ACS) are the major cause of death in Brazil and the world.^1^ Survival rate after an acute coronary event has increased substantially in recent decades, due to advances in drug and reperfusion therapies.^2,3^ However, these patients are still at risk of adverse outcomes such as death, reinfarction, stroke and bleeding.^4^

Anticoagulant therapy is one of the most important steps in the early treatment of ACS. Enoxaparin is traditionally administered with dose titration based on weight, while fondaparinux has a fixed dose.^5^ The OASIS-5 and OASIS-6 studies compared these two drugs in patients with ACS and demonstrated that Fondaparinux is associated with a better outcome, primarily due to a reduction in bleeding events.^6,7^ However, the pharmacokinetics and pharmacodynamics of medications can differ between obese and nonobese patients,^8^ and the fact that obese population have been historically underrepresented in clinical trials leads to uncertainties regarding the application of these results to patients with a BMI ≥ 30 kg/m^2^.

Given the high prevalence of obesity in the global population and its association with an increased risk of coronary artery disease (CAD), establishing an appropriate antithrombotic treatment regimen for these patients is of utmost importance. Thus, the aim of this study was to compare fondaparinux to enoxaparin in the treatment of ACS in a population of obese patients.

## Methods

### Study design

This was a retrospective cohort study of patients hospitalized with ACS at a tertiary care cardiac hospital in Salvador, Bahia, Brazil.

### Study population, inclusion, and exclusion criteria

Patients with a BMI ≥ 30 Kg/m^2^, older than 18 years old, admitted to the hospital with a diagnosis of unstable angina (UA) or Non-ST-Segment Elevation Myocardial Infarction (NSTEMI), and treated with fondaparinux or enoxaparin between 2010 and 2020 were included. Patients transferred to/from another hospital, with type II myocardial infarction (MI), or in whom ACS was ruled out were excluded.

According to institutional protocol, enoxaparin at a dose of 1 mg/kg of weight every 12 hours was the antithrombotic of choice until 2012. Between 2012 and 2019, fondaparinux at a dose of 2.5 mg per day was indicated. After 2018, due to shortage of fondaparinux, enoxaparin was routinely used again.

Patients with impaired renal function (Creatinine clearance, CrCl <30 mL/min/1.73 m²) received a reduced dose of enoxaparin of 1mg/Kg/day.

Patients weighing >100kg received the maximum dose of enoxaparin of 100mg 12/12h; the dose of fondaparinux continued 2.5mg per day.

### Study procedures

Clinical and demographic data, complementary exams, and primary clinical outcomes of all ACS patients were continuously and prospectively collected as part of the quality care program for ACS at the institution during their hospital stay. These data were retrospectively accessed for the study.

### Ethical Considerations

The protocol was approved by the Ethics Committee on Human Research Professor Celso Figueirôa of the Hospital Santa Izabel (Santa Casa de Misericórdia da Bahia) under the number 3.725.420. As it was a retrospective study based on data collected from medical records and institutional quality control records, exemption from informed consent was requested and accepted by the ethics committee.

### Clinical outcomes

The primary outcome consisted of all-cause mortality, reinfarction (based on the 4th universal definition for MI^),^^9^ stroke or major bleeding during hospitalization. Stroke was defined as the presence of a new focal neurological deficit considered of vascular origin with signs or symptoms lasting more than 24 hours. Major bleeding was based on criteria of Hb/Ht ≤ 2g/dL, use of blood transfusion, or hemodynamic instability. Definitions were consistent with those used in the OASIS-5 study.^6^

### Statistical analysis

Data were processed using the Statistical Package for the Social Sciences (SPSS 25.0) program. Categorical variables were described as percentages or proportions and compared using the chi-square test, while continuous variables, as they were normally distributed, were expressed using mean ± standard deviation (SD) and compared using unpaired Student's T-test. The Kolmogorov-Smirnov test and visual histogram analysis were used to verify the normality of the data. The significance level was set at 5%.

Sensitivity analysis was performed with those patients who underwent coronary angiography. We considered this subgroup of patients for sensitivity analysis, due to the risk of bleeding in this population.

## Results

Among 1,578 patients hospitalized for ACS, 367 obese patients with NSTEMI or UA were included, of which 258 used Fondaparinux and 109 used Enoxaparin (Figure 1).

**Figure 1:** Flowchart of the study population selection. BMI: body mass index; NSTEMI: non-ST-segment elevation myocardial Infarction; UA: unstable angina.

In the overall population, mean age was 64 ± 12, 52.9% was male. There were no significant differences between the fondaparinux and enoxaparin groups in terms of clinical characteristics, as shown in Table 1. Only ejection fraction was higher in the enoxaparin group (63 ± 11%) compared to the fondaparinux group (60 ± 13%, p < 0.05), but both were within the normal range. BMI was similar between the groups (33.7 ± 4.0 Kg/m^2^ in fondaparinux versus 33.6 ± 3.5 Kg/m^2^ in enoxaparin). Invasive strategy was performed in 275 patients (74.9%), with no difference between fondaparinux (74.0%) and enoxaparin (77.1%) groups (p = 0.54).

**Table 1:** General characteristics of the study population

	Total (n = 367)	Fondaparinux (n = 258)	Enoxaparin (n = 109)	p value
Age (±SD)	64.5 (±12.4)	63.7 (±12.1)	66.3 (±13.0)	0.08
ACS type				
- Unstable angina n (%)	214 (58.3%)	155 (60.1%)	59 (54.1%)	0.29
- NSTEMI n (%)	153 (41.7%)	103 (39.9%)	50 (45.9%)	0.29
Male Gender n (%)	194 (52.9%)	135 (52.3%)	59 (54.1%)	0.75
Diabetes Mellitus n (%)	162 (44.1%)	115 (44.6%)	47 (43.1%)	0.80
Hypertension n (%)	312 (85.0%)	220 (85.3%)	92 (84.4%)	0.83
Dyslipidemia n (%)	238 (64.9%)	164 (63.6%)	74 (67.9%)	0.43
Smoking n (%)	27 (7.4%)	18 (7.0%)	9 (8.3%)	0.67
Previous CHF n (%)	16 (4.4%)	8 (3.1%)	8 (7.3%)	0.07
Previous PAD n (%)	5 (1.4%)	2 (0.8%)	3 (2.8%)	0.14
Previous CAD n (%)	144 (39.2%)	95 (36.8%)	49 (45.0%)	0.15
Previous PCI n (%)	60 (16.3%)	38 (14.8%)	22 (20.2%)	0.16
Previous CABG n (%)	69 (18.8%)	45 (17.5%)	24 (22%)	0.32
Previous Stroke n (%)	14 (3.8%)	10 (3.9%)	4 (3.7%)	0.93
Killip 1 n (%)	345 (83.9%)	232 (82.6%)	113 (86.9%)	0.26
Medications				
- Aspirin n (%)	349 (95.1%)	248 (96.1%)	101 (92.7%)	0.16
- P2Y12 receptor inhibitors n (%)	340 (92.6%)	243 (94.2%)	97 (89.0%)	0.08
- Abciximabn (%)	5 (1.4%)	4 (1.6%)	1 (0.9%)	0.63
- Tirofiban n (%)	1 (0.3%)	0 (0.0%)	1 (0.9%)	0.12
- NOAC n (%)	11 (3.0%)	5 (1.9%)	6 (5.5%)	0.07
- Warfarin n (%)	7 (1.9%)	3 (1.2%)	4 (3.7%)	0.11
Invasive Strategy n (%)	275 (74.9%)	191 (74.0%)	84 (77.1%)	0.54
PCI n (%)	92 (25.9%)	66 (26.4%)	26 (24.5%)	0.66
Radial Access n (%)	220 (59.9%)	151 (58.5%)	69 (63.3%)	0.39
Average SBP (±SD) mmHg	145.6 (±25.4)	146.9 (±25.8)	142.6 (±24.3)	0.14
Average HR (±SD) bpm	74.3 (±16.4)	73.5 (±15.4)	76.0 (±18.5)	0.19
Average Creatinine (±SD) mg/dl	1.0 (±0.34)	0.99 (±0.32)	1.03 (±0.39)	0.23
Creatinine Clearance (±SD) mL/min/1.73m²	98.3 (±29.2)	99.8 (±28.3)	94.7 (±31.2)	0.12
Average Ejection Fraction (±SD) %	0.62 (±0.11)	0.63 (±0.11)	0.60 (±0.13)	0.02
Average Hb(±SD) mg/dl	13.6 (±1.5)	13.6 (±1.5)	13.5 (±1.6)	0.65
Average Ht (±SD) %	41.0 (±4.4)	41.1 (±4.3)	40.8 (±4.6)	0.49
Average BMI (±SD) kg/m^2^	33.7 (±3.8)	33.7 (±4.0)	33.6 (±3.5)	0.81
Average Length of hospitalization (±SD) in days	6.1 (±6.5)	5.8 (6.4)	6.8 (6.7)	0.18

ACS: acute coronary syndrome; NSTEMI: non-ST-segment elevation myocardial infarction; CHF: congestive heart failure; PAD: peripheral arterial disease; CAD: coronary artery disease; Stroke: stroke; NOAC: new oral anticoagulants; SBP: systolic blood pressure; HR: heart rate; SD: standard deviation; Hb: hemoglobin; Ht: hematocrit; BMI: body mass index; PCI: percutaneous coronary intervention; CABG: coronary artery bypass grafting.

The incidence of the primary outcome (death, reinfarction, stroke or major bleeding) was similar between the groups: 4.7% in the fondaparinux group, and 5.5% in the enoxaparin group (p = 0.729) (Figure 2). Furthermore, there was no significant difference between the groups regarding the secondary outcome components, both when analyzed together and individually.

**Figure 2:** Incidence of primary outcomes in the study population. reAMI: re-myocardial infarction.

Sensitivity analysis is demonstrated in Figure 3. The incidence of the primary composite endpoint remained similar between groups when analyzed only those patients who underwent invasive strategy: 5.8% in the fondaparinux group, and 7.1% in the enoxaparin group (p = 0.661). There was also no significant difference between groups regarding the composite outcome of death, reinfarction or stroke (p = 0.135), or when the endpoints were analyzed individually (Figure 3).

**Figure 3:** Incidence of primary outcomes in patients who underwent coronary angiography (sensitivity analysis). reAMI: re-myocardial infarction.

## Discussion

Based on a retrospective cohort study of patients admitted for UA or NSTEMI, our study compared fondaparinux with enoxaparin in the treatment of obese patients. No difference was identified in the incidence of death, reinfarction, stroke or major bleeding between the two subgroups. It is hypothesized that fondaparinux is as effective and safe as Enoxaparin, even in obese patients, without the need for dose adjustment.

The OASIS-5 study^6^ evaluated a sample of 20,078 patients with NSTEMI randomized to fondaparinux or enoxaparin, identifying equivalence between the strategies in the primary study outcome (death or myocardial infarction or refractory ischemia in nine days). A reduction in the occurrence of major bleeding was identified in those using fondaparinux (2.2% versus 4.1%, RR 0.52; p < 0.001), resulting in a reduction in 30-day mortality in favor of fondaparinux. However, the study did not specifically evaluate patients with BMI ≥ 30 kg/m^2^, leaving the question of whether the fixed dose of 2.5 mg of fondaparinux would have a similar performance in patients in this BMI range.

Soeiro et al.^10^ conducted a multicenter retrospective cohort study including a total of 2,282 patients and compared Fondaparinux to Enoxaparin based on a Brazilian registry. Similarly to the OASIS trials, Fondaparinux proved superiority to Enoxaparin for the Brazilian population, with significant reduction of bleeding and combined events (cardiogenic shock, MI, death, ischemic stroke, and major bleeding). There was no difference in the primary outcome of in-hospital all-cause mortality between the two groups.

In our analysis, there was no significant difference in the incidence of bleeding events between obese patients using fondaparinux and enoxaparin. Different from what was found in previous studies, there was a tendency towards a higher bleeding rate in the Fondaparinux group (2.3% x 0.9%). The fact that this difference was not statistically significant (p=0.368) may be related to the reduced power in analyzing this outcome alone. To our knowledge, there is no other study in the literature evaluating the safety and efficacy of fondaparinux in obese patients with non-ST-elevation ACS. Still in the context of ACS, Spinler at al.^11^ performed a subgroup analysis (obese vs. nonobese) based on the combined data of the ESSENCE and TIMI-11B clinical trials. These randomized studies compared enoxaparin to unfractionated heparin (UFH) for the treatment of ACS. The primary endpoint used in the combined analysis was the composite end point of death, MI and urgent revascularization, and the superiority of enoxaparin over UFH was observed in both the non-obese and obese subgroups. Although this study has different interventions and comparisons, the favorable result in the obese population corroborates our present study, suggesting that there is no need of adjustment to therapy in obese patients.

Davidson et al.,^12^ based on MATISSE trials data, compared the use of Fondaparinux to the classical treatment with heparins (Enoxaparin or UFH) in obese and non-obese patients with venous thromboembolism (deep vein thrombosis and pulmonary embolism), and demonstrated that, regardless of BMI, there was no differences in the rate of bleeding between the groups fondaparinux and heparins. Although this study was conducted in a different population (venous thromboembolism), these findings corroborate our findings, suggesting that the BMI ≥30 Kg/m^2^ has little or no impact on the safety and efficacy of anticoagulant therapy. Thus, our data support the use of Fondaparinux at its usual dose for ACS (2,5mg) in patients with BMI ≥ 30 Kg/m^2^.

This research is pioneering in comparing clinical outcomes related to the use of Fondaparinux or Enoxaparin in obese patients with ACS. However, some methodological limitations should be highlighted, primarily the observational and retrospective nature of the study, although the clinical characteristics of the populations were similar. A propensity score analysis could be used in studies like ours, however, as the baseline characteristics were well balanced between groups, we chose not to perform it. In addition, as the population was limited to obese patients, our study sample size was small, which may have masked eventual differences between the groups. Furthermore, in clinical outcome, we could only assess all-cause death, and would be of interest to separate it from cardiovascular deaths. Another important point is that we had a convenience sample.

Furthermore, we believe that our study brings significant value in generating hypotheses, and prospective and randomized studies in this population are warranted to confirm these findings. Studies with a cohort of patients with non-ST-elevation ACS using fondaparinux, including the comparison of effectiveness and safety between obese and non-obese, would be of interest.

## Conclusions

In a sample of patients with BMI ≥ 30Kg/m^2^ and ACS without ST-segment elevation, antithrombotic treatment with Fondaparinux or Enoxaparin was not associated with differences in the occurrence of major cardiovascular events or major bleeding during the hospitalization period.
